# Direct Observation of the Exciton-Polaron in Single CsPbBr_3_ Quantum Dots

**DOI:** 10.1021/acsnano.5c06716

**Published:** 2025-08-02

**Authors:** Zhou Shen, Margarita Samoli, Onur Erdem, Johan Bielecki, Amit K. Samanta, Juncheng E, Armando D. Estillore, Chan Kim, Yoonhee Kim, Jayanath Koliyadu, Romain Letrun, Federico Locardi, Jannik Lübke, Abhishek Mall, Diogo V. M. Melo, Grant Mills, Safi Rafie-Zinedine, Adam Round, Tokushi Sato, Raphael de Wijn, Tamme Wollweber, Lena Worbs, Yulong Zhuang, Adrian P. Mancuso, Richard Bean, Henry N. Chapman, Jochen Küpper, Ivan Infante, Holger Lange, Zeger Hens, Kartik Ayyer

**Affiliations:** † 375070 Max Planck Institute for the Structure and Dynamics of Matter, 22761 Hamburg, Germany; ‡ Physics and Chemistry of Nanostructures, 26656 Ghent University, Gent 9000, Belgium; § 339694 European XFEL, 22869 Schenefeld, Germany; ∥ Center for Free-Electron Laser Science CFEL, 28332 Deutsches Elektronen-Synchrotron DESY, 22607 Hamburg, Germany; ⊥ Dipartimento di Chimica e Chimica Industriale, 9302 Università degli Studi di Genova, 16146 Genova, Italy; # The Hamburg Center for Ultrafast Imaging, 22761 Hamburg, Germany; ∇ Department of Chemistry and Physics, La Trobe Institute for Molecular Science, La Trobe University, Melbourne, VIC 3086, Australia; ° Department of Physics, University of Hamburg, 22761 Hamburg, Germany; ◆ 518636 BCMaterials, Basque Center for Materials, Applications, and Nanostructures, UPV/EHU Science Park, Leioa 48940, Spain; ¶ Ikerbasque Basque Foundation for Science, Bilbao 48009, Spain; †† University of Potsdam, Institute of Physics and Astronomy, 14476 Potsdam, Germany; ‡‡ NoLIMITS Center For Non-Linear Microscopy and Spectroscopy, Ghent University, Gent 9000, Belgium

**Keywords:** quantum dots, polarons, ultrafast, X-ray diffraction, XFEL

## Abstract

The Outstanding optoelectronic properties of lead halide perovskites have been related to the formation of polarons. Nevertheless, the observation of the atomistic deformation brought about by one electron–hole pair in these materials has remained elusive. Here, we measure the transient structure of single CsPbBr_3_ quantum dots (QDs) after resonant excitation in the single exciton limit using serial femtosecond crystallography (SFX). By reconstructing the three-dimensional (3D) differential diffraction pattern and building on density functional theory (DFT) calculations, we assign the lattice distortion after photoexcitation to the combined presence of a delocalized electron and a localized hole, forming a mixed large/small exciton-polaron. This result creates a clear picture of the polaronic deformation in CsPbBr_3_ QDs and demonstrates the exceptional sensitivity of SFX to lattice distortions in few-nanometer crystallites. We plan to use this experimental platform for future studies of electron–lattice interactions.

## Graphical abstract

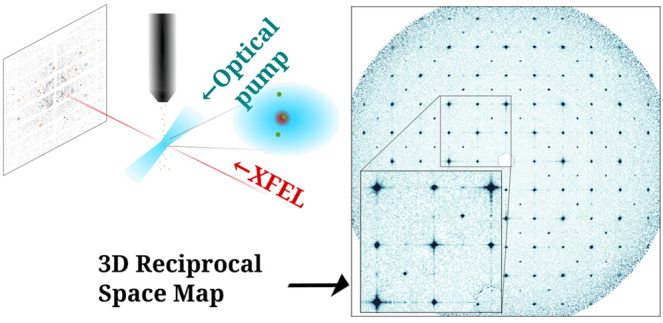

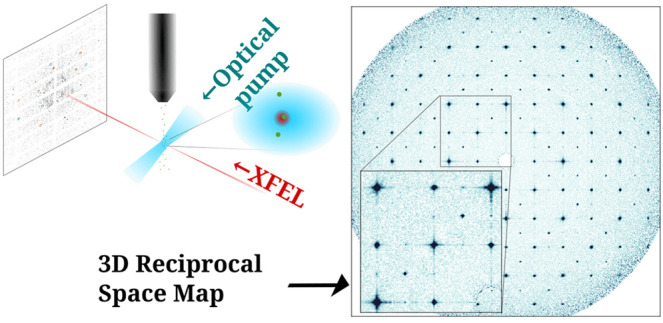

A polaron is a quasi-particle in a crystalline lattice consisting of a material object, such as an electron, and an accompanying lattice deformation field. Large polarons have deformation fields extending well beyond a single unit cell, while small polarons involve a more localized lattice distortion.^1^ In particular, in the case of lead halide perovksites (LHPs), which developed in recent years from an absorber material in highly efficient solar cells to a multipurpose semiconductor for detecting and emitting light,^2,3^ experimental and computational studies have related specific optoelectronic characteristics to polaron formation.^4^ Large polarons, for example, have been linked to the enhanced charge-carrier lifetime,^5^ long diffusion length,^6^ and slow second-order electron–hole recombination.^7^ However, as many studies on polarons in LHPs rely on computational methods for their interpretation, the need remains for experimental verification of the role polarons play in charge transport in LHPs:^1^ a task hampered by the lack of a direct observation of the polaron-related lattice distortion.

Over the last 5 years, several studies have investigated changes in the atomic lattice of LHP nanocrystals (NCs) after optical pumping with femtosecond (fs) or picosecond (ps) time resolution using pulsed X-ray or electron probes. For such studies, NCs have the advantage of hosting well-defined optical excitations, such as strongly confined electron–hole pairs or bound two-dimensional excitons.^8,9^ Using 80 ps X-ray pulses, for example, 11.2 nm CsPbBr_3_ NCs were shown to undergo heating-induced phase transitions,^10^ while two-dimensional (2D) LHPs exhibited an anisotropic lattice expansion.^11^ Furthermore, a rapid, subpicosecond buildup of lattice distortions was observed on 10 nm CsPbBr_3_ NCs by means of femtosecond electron diffraction,^12^ while the same method was used to estimate the electron–phonon coupling strength in these materials.^13^ However, while showing the potential of optical pump/diffractive probe methods to analyze photoinduced changes in the crystal structure, these studies invariably used nonresonant optical pumping at power densities that created multiple excitations per NC, and which analyzed azimuthally averaged diffraction profiles. To observe the atomic lattice distortion caused by a single electron–hole pair, the key characteristic of an exciton-polaron in such systems, both excess heat and polarization-field overlap must be avoided. Such conditions require resonant excitation at power densities that create only a single excitation per NC, presumably in combination with a 2D or three-dimensional (3D) reciprocal space map of the light-on–light-off differential diffraction.

In this study, we use the light-on/light-off diffraction difference of femtosecond X-ray pulses generated by a free-electron laser (XFEL) to determine the deformation field in 4.9 nm cubic CsPbBr_3_ NCs after resonant excitation. XFELs have been used to study deviations from crystalline order at ultrafast time scales either on single crystals^14,15^ or powders,^16,17^ including on LHP single crystals.^18^ However, to be sensitive to the small deformation field of single exciton-polarons, we moved from analyzing NC powders to analyzing them by serial femtosecond crystallography (SFX). In SFX, one snapshot at a time is taken on a series of single NCs with and without photoexcitation, for which we used a fixed pump/probe delay. Inspired by structural studies of small-molecule systems^19,20^ and ultrafast dynamics of proteins,^21–23^ we reconstructed the 3D diffraction pattern in reciprocal space by indexing the observed Bragg peaks for each NC to determine the crystal orientation.^24^ When obtained from a probe that is coherent across the entire crystal, such a 3D diffraction map is exquisitely sensitive to lattice distortions. Picometer sensitivities have been reported, for example, using the Bragg coherent diffractive imaging technique.^25^ The principle is illustrated in Figure 1a,b. Here, red and green dots represent atomic columns in real space of a small cubic NC of a fictitious diatomic compound with a body-centered structure. Figure 1a shows an NC as cut from the bulk and the corresponding diffraction map sliced normal to the ⟨001⟩ axis. Each Bragg peak is convolved with the so-called shape transform, which is the Fourier transform of a 3D mask that is 1 inside and 0 outside the NC. In Figure 1b, a radial deformation is added to the NC, which is different in direction for the two sublattices. This deformation field changes the diffraction map, which leads to a differential diffraction for each Bragg peak, as shown in Figure 1b.

**Figure 1:**
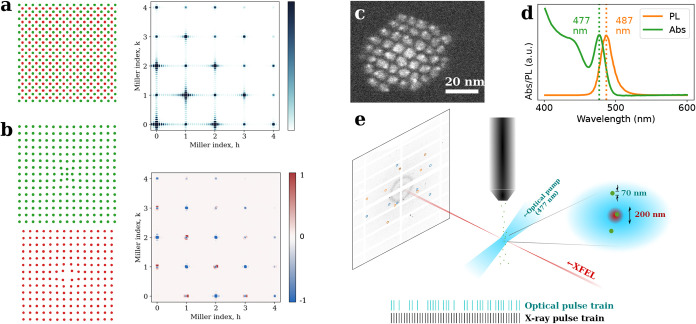
Toy model and experimental schematic. (a) Toy model. Illustration of a diatomic body-centered cubic NC with the associated 3D diffraction map in the *hk* plane. (b) Lattice distortion of the two sublattices and the resultant differential diffraction map (positive differences in red). (c) Representative transmission electron microscope image and (d) absorbance and photoluminescence (PL) spectra of the CsPbBr_3_ NCs used in this study. (e) Experimental setup. Aerosolized particles are intercepted by the XFEL beam to produce diffraction patterns on the detector. Half of the pulses are preceded by an optical pump pulse with a pump–probe delay of 3 ps. The pattern of pulses with an excitation within the first 50 pulses of the European XFEL pulse train is illustrated at the bottom. The inset shows the interaction region with a 200 nm XFEL focus intercepting ∼70 nm droplets consisting of NCs and nonvolatile buffer components.

## Results

### Time-Resolved Aerosol Serial Femtosecond Crystallography

We synthesized a batch of CsPbBr_3_ NCs using previously published protocols based on size-selective precipitation.^26^ As evidenced by the dark field scanning transmission electron microscopy (DF-STEM) image and the absorption spectrum shown in Figure 1c,d, these NCs have an average diameter of 4.9 nm and exhibit an exciton transition at 477 nm. Since the shift of this transition with respect to bulk CsPbBr_3_ has been assigned to partial confinement of charge carriers,^9^ we will refer to these NCs henceforth as quantum dots (QDs). In line with previous studies,^27^ the exciton emission has a maximum intensity at 487 nm, which corresponds to a 53 meV Stokes shift. As outlined in Figure 1e, we exposed these QDs a few at a time to an XFEL pulse by means of an aerosol sample delivery system that was originally developed for imaging single biomolecules.^28^ Upon aerosolization, the nonvolatile components of the solvent mixture formed a 70 nm droplet, which contained on average 1.6 QDs each. In SFX, the measurements are performed in the diffraction-before-destruction regime^29^ where each NC is measured only once and is destroyed after exposure. The ultrashort pulse duration (<50 fs) means that diffraction can be measured before radiation damage sets in.^30^ Through this delivery method, the sample is refreshed after each exposure, while avoiding the strong background scattering produced by liquid jets commonly used for SFX on protein crystals. To the best of our knowledge, a similar approach has only been reported for NCs with dimensions of a few 100 nm,^24,31^ and for some fiber crystals with diameters of tens of nanometers, or longer.^32,33^

Figure 2a represents a typical diffraction snapshot containing Bragg spots recorded as part of a sequence of 77,182 frames (detailed statistics in Supporting Information S1). In this particular frame, we identified diffraction from three different QDs, as indicated by the colored circles. Throughout the experiment, half the frames were collected 3 ps after resonant excitation of the QDs, which is long enough for any lattice distortion to settle^13^ and short enough to prevent heat generation from biexciton recombination.^9^ Using a 120 fs optical pulse with a wavelength of 477 nm and a fluence of 67.5 μJ/cm^2^,^34^ we expect to probe one electron–hole pair per QD in ≈75% of the pumped frames; see Supporting Information S2. Unpumped and pumped snapshots were examined for Bragg peaks and considered hit when they contained at least two identified peaks. For each hit, Bragg peaks were indexed in the orthorhombic *Pnma* space group and assigned to one or more QDs as illustrated by the colored circles in Figure 2a (see Methods section for details). In total, 31,547 crystals were indexed from 20,103 frames with detected peaks.

**Figure 2:**
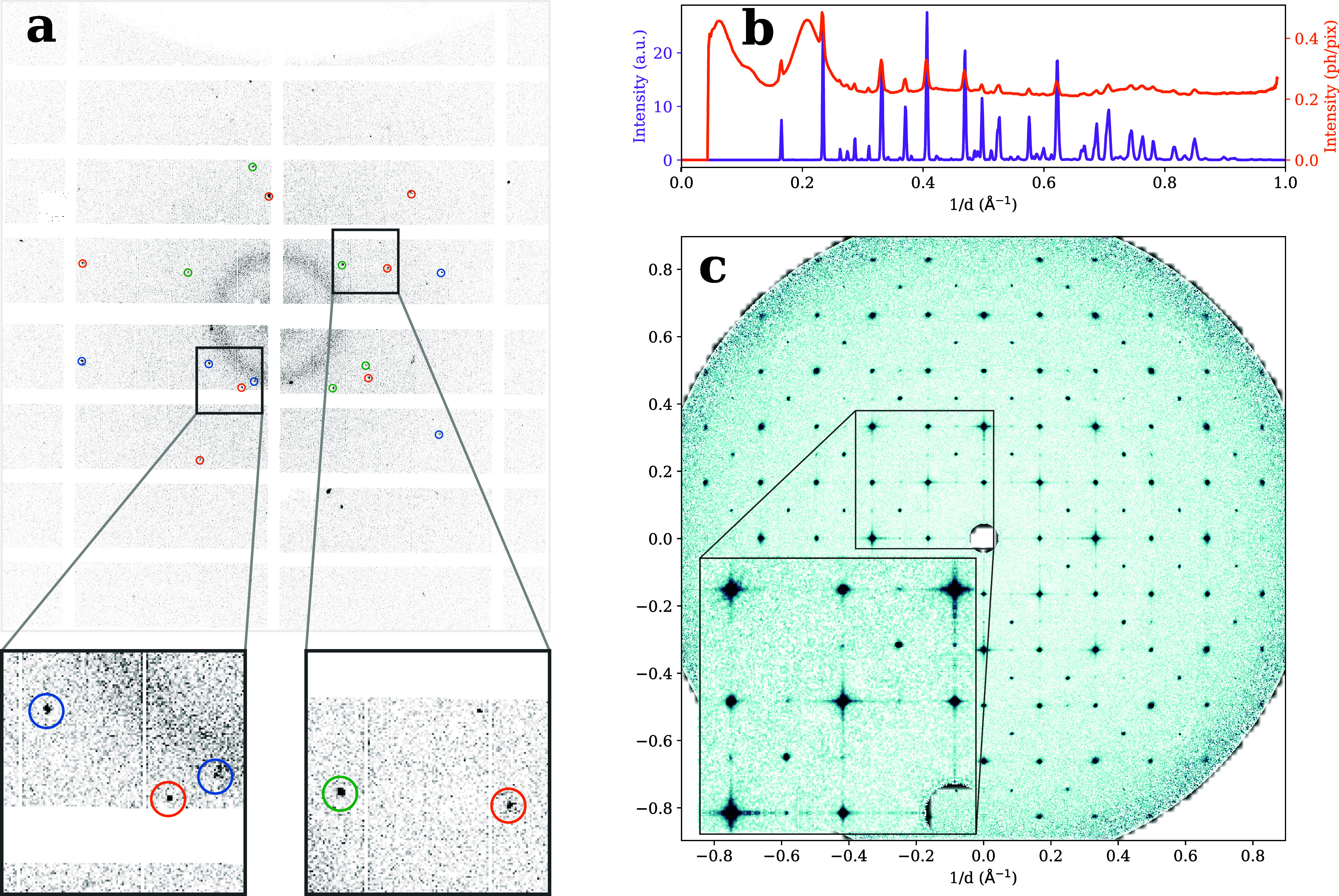
Diffraction data and reconstruction. (a) Example of a 3-crystal indexed pattern. The peaks of each crystal are circled by different colors. The diffuse ring corresponds to the signal from the nonvolatile components of the buffer such as the ionic liquid. (b) Virtual powder plot in purple calculated by integrating Bragg peak intensities after peak finding from individual patterns. Note that by only including the signal from peaks, one avoids contributions from the diffuse background and obtains peaks sharper than the peak width on the detector. The azimuthally averaged intensity calculated directly from the detector frames, analogous to a conventional powder diffraction measurement, is shown in orange. One can observe the diffuse background due to the nonvolatile solvent such as the ionic liquid and the relative suppression of high-angle peaks. (c) Central slice (*q*_*x*_ = 0) of the average dark (unpumped) intensity obtained by merging whole patterns according to the orientations predicted by indexing the peaks in 44000 patterns after appropriate diffuse background subtraction. The inset shows a subregion with an expanded color scale to visualize the peak shapes and tails.

From the indexed snapshots, the azimuthally averaged virtual powder pattern can be recovered in two ways, as illustrated in Figure 2b. The orange trace is the azimuthal average ⟨*I̅*(*q*)⟩ of all snapshots, where *q* is the magnitude of the reciprocal wave vector ***q*** = (*q*_*x*_, *q*_*y*_, *q*_*z*_), expressed as the reciprocal of the *d*-spacing. This intensity corresponds to the quantity that would be obtained from conventional powder diffraction. The purple trace, on the other hand, represents the intensity *I̅*(*q*) of the Bragg peaks, averaged over all hits after a single-shot peak finding. Clearly, the serial recording of diffraction snapshots of single QDs strongly enhances the measurement sensitivity. In particular, the diffuse background at all *q*, and a broad but pronounced feature at around 0.2 Å^–1^ are almost entirely removed in *I̅*(*q*). We separately merged the integrated peak intensities from the diffraction snapshots recorded without and with optical pumping and used the resulting patterns *I̅*_dark_(*q*) and *I̅*_pump_(*q*) to solve for the average unit cell using the SHELXL software to 1.1 Å resolution.^35^ However, no statistically significant difference between the two patterns was observed in either the integrated peak intensities, the virtual powder patterns, or the azimuthal average intensity. This observation agrees with a previous report, where no change in the electron diffraction powder pattern was observed on 9.5 nm CsPbBr_3_ NCs excited using 400 nm pulses with a fluence of 800 μJ/cm^2^, 12-fold of what was used in the experiments reported here.^13^

More interestingly, having an indexed series of diffraction snapshots of single QDs, and thus the QD orientation, enabled us to move beyond azimuthal averaging and reconstruct an average diffraction pattern *I̅*(***q***) in 3D reciprocal space. To do so, we masked the region around Bragg peaks from other QDs in the same detector frame and subtracted a scaled diffuse background for each indexed QD before merging all pixels (see Methods section for details). Figure 2c displays a central slice of the resulting dark pattern normal to the *q*_*x*_-axis. Various features can be qualitatively identified in this intensity distribution. The square grid made up by the bright Bragg peaks is characteristic of an approximately cubic lattice with an average lattice constant of 5.90 Å, while additional weak peaks can be observed at half-integer positions in line with previous orthorhombic crystal structure assignments.^36^ In the rest of this article, we will use the effective cubic lattice peaks with Miller indices (*h*_C_, *k*_C_, *l*_C_). The bright peaks also exhibit lattice truncation streaks along the ⟨100⟩ directions due to the approximately cubic shape of individual QDs. Finally, the intensity distribution of the Bragg peaks is somewhat asymmetric around the reciprocal lattice point, which probably reflects inherent strain in these QDs even before optical excitation. We do not observe fringe contrast along the ⟨100⟩ streaks because the formation of the merged pattern involves averaging over QDs with slightly different sizes and inherent strain.

### Optically Induced Lattice Deformations

Figure 3a displays a slice of the diffraction intensity difference in the *q*_*x*_–*q*_*y*_ plane, Δ*I̅*(*q*_*x*_, *q*_*y*_, *q*_*z*_ = 0), obtained by subtracting *I̅*_dark_ from *I̅*_pump_. Interestingly, while the virtual powder patterns *I̅*_pump_ and *I̅*_dark_ were similar, Bragg peaks in this differential diffraction pattern show an intricate combination of enhanced (red) and reduced (blue) intensities as a result of optical pumping. Especially for the (200) and (110) diffraction, these patterns can be interpreted in the first approximation as an inward or outward shift of the Bragg peak, respectively. Given the direct link between the diffraction pattern and the atomic structure, we thus conclude that the resonant formation of a single electron–hole pair in 4.9 nm CsPbBr_3_ QDs comes with a distortion of the atomic lattice, i.e., the formation of an exciton-polaron.

**Figure 3:**
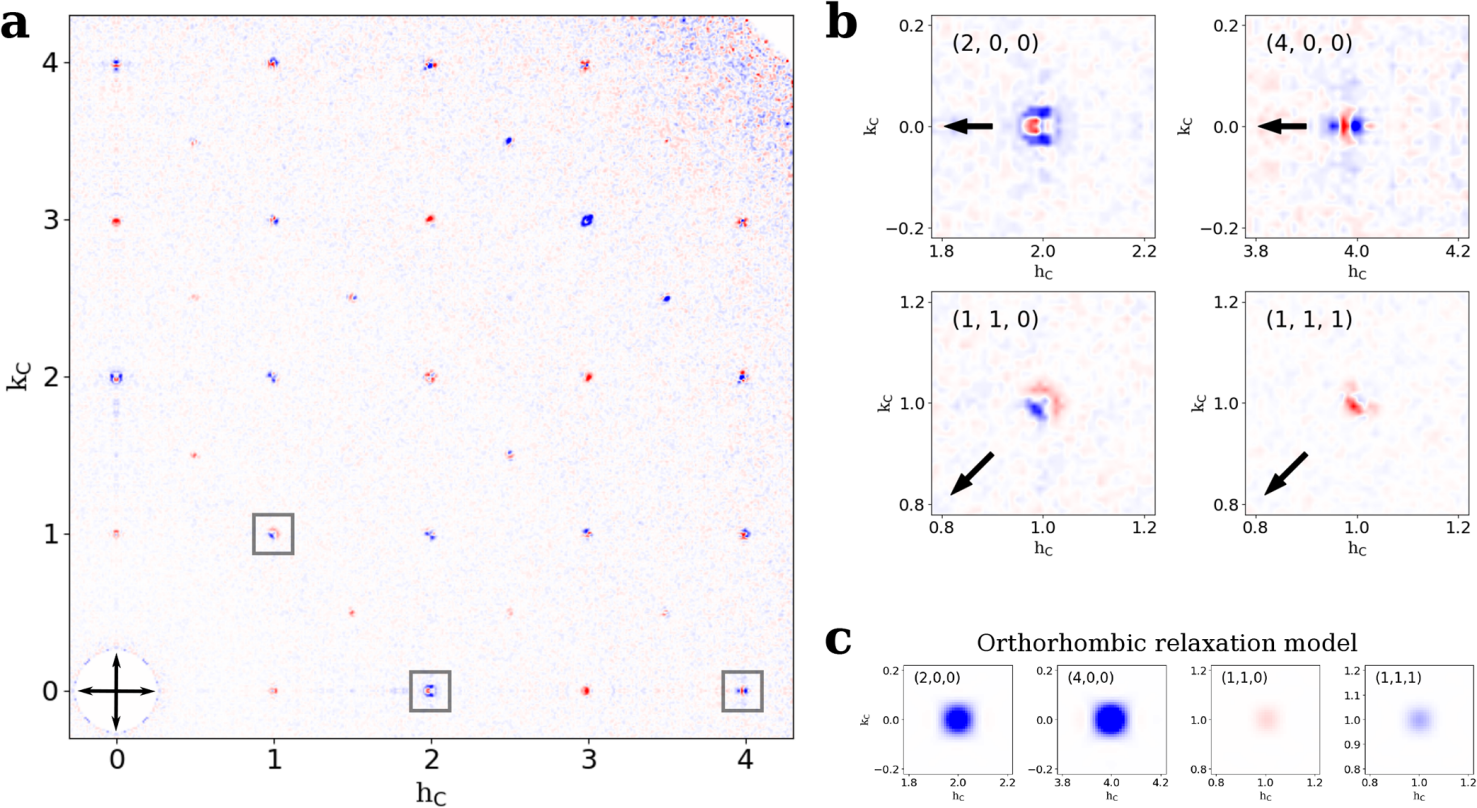
Difference intensities upon optical excitation. (a) Slice through the *l*_C_ = 0 plane of the merged intensity difference before and 3 ps after optical excitation. Values in red indicate regions with excess intensity after optical pumping. (b) Expanded view of four selected Bragg peaks (arrows point toward **q** = 0), three of which are highlighted in panel (a). For the (111) peak, *l*_C_ = 1. (c) Predicted intensity differences from a commonly applied orthorhombic relaxation model where the octahedral tilt varies across the crystal. Note how this distortion does not result in peak shifts but just changes in integrated intensities.

Qualitative observations of differential diffraction provide a first understanding of the lattice deformation field. First, difference scattering signals in Figure 3a are concentrated near the Brillouin zone (BZ) center, indicative of long-range correlations in the deformation field. A strongly localized lattice distortion will produce difference scattering throughout the reciprocal space, but this is not visible in the data. In fact, outside of the crystal truncation streaks resulting from the cubic shape of the NCs, no diffuse scattering is observed far from the BZ center. Second, the differential diffraction appears to be primarily in the radial direction and not along, for example, the ⟨100⟩ facets. This finding is highlighted by the differential diffraction of four representative peaks in Figure 3b, where the arrow represents the direction toward the origin. While Figure 3a shows intensity differences in the neighborhood of all peaks, we focus on four of the strongest reflections where the peak shifts are reproducible (Supporting Information S5). The fractional intensity differences for these four reflections are described in Supporting Information S3. The positions of the maxima in the difference images for the 200, 400, and 110 peaks are 2% of the cubic reciprocal lattice spacing (1/*d* = 0.003 Å^–1^) and half of that for the 111 peak. Such systematics suggest a predominantly spherically symmetric deformation field, although the presence of off-axis or tangential features means that the deformation field is not perfectly isotropic.

A commonly proposed deformation field involves a relaxation of the orthorhombic distortion of the Br octahedra upon optical excitation;^12,13,37^ see Supporting Information S4. Figure 3c displays the differential diffraction such a distortion would cause (see Methods section for details). Interestingly, this deformation field yields a differential diffraction that mostly reflects small changes in the integrated diffraction intensity rather than the experimentally observed shifts of the Bragg peaks. It thus appears that a single exciton-polaron created through resonant excitation leads to a different lattice distortion than multiple electron–hole pairs formed by nonresonant excitation.

### Density Functional Theory Modeling

A 3D diffraction difference map is a rich source of information that encodes the deformation field ***u*** in reciprocal space. However, the differential diffraction measured here results from subtracting light-on/light-off diffraction intensities calculated as separate averages over an ensemble of differently sized QDs and cannot be used for the *ab initio* reconstruction of the deformation field. To create a first benchmark relating the experimental differential diffraction to the deformation field of the exciton-polaron in CsPbBr_3_ QDs, we calculated the atomistic structure of CsPbBr_3_ QDs using density functional theory (DFT). As outlined in the Methods section, we relaxed the geometry of ≈3 nm CsPbBr_3_ QDs with the brute formula Cs_200_Pb_125_Br_450_ in the *S* = 0 singlet ground state and the *S* = 1 triplet excited state. While the latter yields the triplet state of the exciton, we assumed that a spin flip of the exciton has no significant impact on the atomic geometry. For both states, we obtained the coordinates of different atoms and the densities of valence electrons. From these data, we calculated diffraction intensity differences that can be directly compared with the corresponding slices of the experimental 3D differential diffraction. A full overview of the DFT approach and results is provided in Supporting Information S6.

Figure 4a represents the relaxed structure of one of the CsPbBr_3_ QD models, which are cut as cubes from the bulk CsPbBr_3_ lattice. To ensure charge neutrality, we removed the 8 vertex Cs atoms and 8 additional edge Cs atoms. Five models were considered for which different edge Cs atoms were removed. Averaged over these 5 models, we obtained a mean relaxation energy when optimizing the geometry of the *S* = 1 triplet excited state starting from the *S* = 0 singlet ground state of 56 meV. This number would correspond to a Stokes shift of 112 meV, which exceeds the experimentally observed shift of 53 meV for 4.9 nm CsPbBr_3_ QDs, but is comparable to extrapolated shifts for 3 nm QDs.^27^

**Figure 4:**
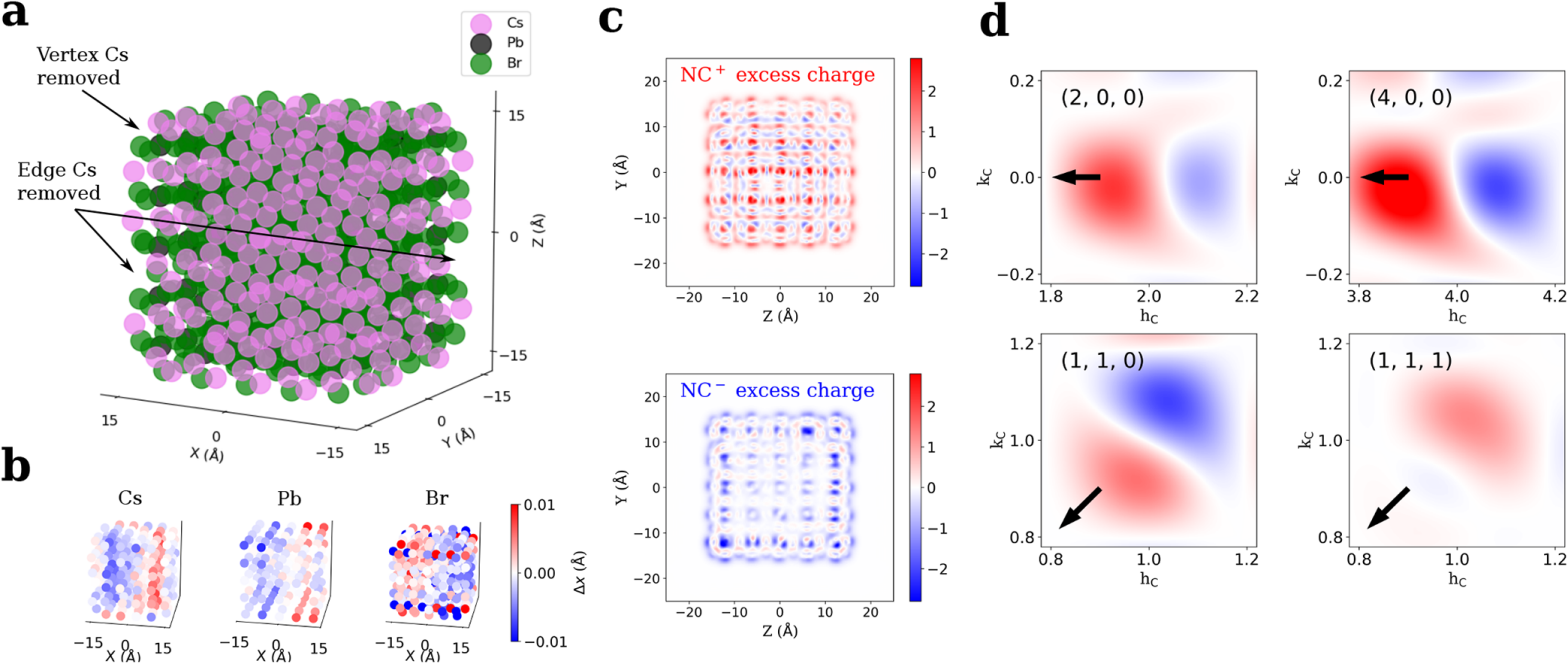
Density functional theory (DFT) modeling. (a) Representation of one of the constructed 3 nm QDs. Some of the 16 Cs atoms removed for charge neutrality are indicated. (b) Atomic displacements along the *x* direction for the different sublattices between the relaxed triplet and singlet states. Other than the outermost layer, one can visualize the outward displacements of Cs and Pb atoms and inward displacement of Br atoms. (c) Excess charge density maps for the cationic and anionic NCs, respectively, projected along the *x*-axis, showing the localization of the hole and excess negative charge concentrated in the outer regions. (d) Predicted intensity difference for the same peaks as in Figure 3b. Note the broader peaks due to the 3 nm simulated particle.

Next, we obtained ***u*** from the difference in relaxed atomic positions of the *S* = 1 excited state and the *S* = 0 ground state. Figure 4b shows *u*_*x*_—the *x*-component of the displacement—of the three atomic sublattices for the CsPbBr_3_ QD model shown in Figure 4a. Here, atoms are colored red or blue depending on *u*_*x*_ being positive or negative. While the relaxation is fairly complex, this color coding underscores that, on average, Cs and Pb cations move outward—positive shift at the right side and negative shift at the left side of the QD—while Br anions move inward. Combining all field components yields, to the first approximation, a radial deformation field with outward shifts for the cations and inward shifts for the anions (see Methods section for details). Such a longitudinal field is consistent with a charge distribution that involves a central positive charge and a distributed negative charge. This conclusion is illustrated by the projected excess charge maps obtained by removing or adding an electron to the CsPbBr_3_ QD (see Figure 4c) and is consistent with recent spectroscopic evidence obtained on the same system.^9,38^ Furthermore, the differential diffraction calculated from the *S* = 1 excited and the *S* = 0 ground state yields a pattern of shifted Bragg peaks that point toward the center of reciprocal space; see Figure 4d. Even so, while calculated and experimental differential diffractions are consistent around the 200 Bragg peak, they are not consistent around 110 and 111. For those peaks, the predicted inward shift (Figure 4d) contrasts with a measured outward shift (Figure 3b). We thus conclude that DFT calculations on 3.0 nm CsPbBr_3_ QDs do not fully grasp the average structure of the exciton-polaron in 4.9 nm CsPbBr_3_ QDs.

### Random Hole Localization Model

The DFT calculations yielded a mostly radial deformation field ***u***, where cations shifted outward and anions inward, in agreement with the electric field of a charge distribution involving a localized positive and a delocalized negative charge. We took such a presumed exciton charge distribution as a starting point to get an improved estimate of ***u*** from the balance between the electric force and the restoring elastic force on each atom. Considering the restoring force constant as an adjustable parameter, we tuned the relative displacement of different atoms and compared the predicted and experimental diffraction differences. However, no matter the ratio of the relative displacement amplitudes, a central positive charge never yielded an outward shift of the 110 peaks using this approach (see Supporting Information S7). We therefore concluded that the experimental differential diffraction is not the mere average of exciton-polarons characterized by a central positive charge over an ensemble of photoexcited QDs.

As can be seen in Figure 4c, removing one electron from a CsPbBr_3_ QD yields an excess positive charge that is somewhat off-center and has a considerable surface contribution. We conjectured that this deviation from a central charge will be more pronounced for the larger QDs that we analyzed experimentally. Hence, to better describe the average deformation field of many QDs, we calculated the diffraction from an ensemble of pumped QDs with hole charges randomly localized within the QD. The displacement of each atom is then proportional to the net field from a point positive charge and a delocalized negative charge; see Figure 5a. This approach consistently yielded inward shifts of the 200 Bragg peak, in agreement with the experimental differential diffraction. Furthermore, by introducing a bias that favors hole localization closer to the QD surface (illustrated in Figure 5b), we can reproduce the experimentally observed combination of an inward shift of the 200 and an outward shift of the 110 Bragg peak; see Figure 5c. This approach also captures the main characteristics of the 400 and 111 Bragg peaks. By scanning the relative displacement field amplitudes (see Supporting Information S7), we also observe that the correct peak shift for the 200 peak requires that the Br atoms are more weakly restrained than the Cs and Pb atoms. This result indicates that upon photoexcitation, an exciton-polaron is formed that consists of a delocalized electron and a localized hole, the position of which is biased toward the outer parts of the QD. This charge distribution induces atomic displacements proportional to the local electric field and the ionic charge, with Br atoms displacing more than the Cs and Pb atoms.

**Figure 5:**
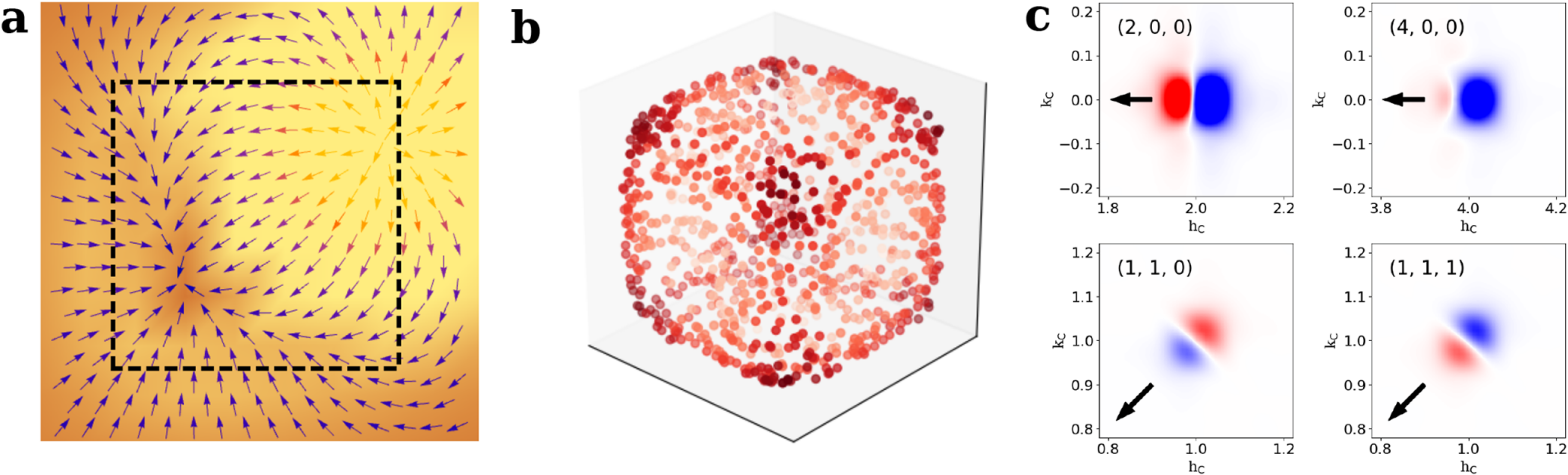
Random hole localization. (a) Electric field distribution in the central plane resulting from a localized hole near the surface and a delocalized electron density. The dashed line represents the boundary of the NC. (b) Sampled hole positions in the volume of the CsPbBr_3_ NC (color represents the distance from the center). (c) Predicted average intensity difference for the same peaks as in Figure 3b calculated by averaging the sampled hole positions in panel (b).

## Conclusions

By means of serial femtosecond crystallography, we reconstructed the 3D diffraction pattern in the reciprocal space of 4.9 nm CsPbBr_3_ QDs. Comparing light-on/light-off diffraction, we obtained differential diffraction patterns that featured subtle changes after photoexcitation that are not resolved in the azimuthally averaged powder pattern. More precisely, the 3D differential diffraction map in reciprocal space shows a radial pattern that reflects, most simply, inward or outward shifts of the Bragg peaks. Supported by density functional theory calculations, we argue that in real space, these shifts reflect the formation of an exciton-polaron, which distorts the lattice by displacing cations outward and anions inward. This distortion resembles a longitudinal deformation brought about by the electric field of a localized positive and a delocalized negative charge. A more detailed analysis indicates that the opposite shifts of the 200 and 110 Bragg peaks result from the ensemble averaging—intrinsic to the SFX approach—of exciton-polarons having a different, surface-biased hole localization. Interestingly, in agreement with the partial confinement of charge carriers in CsPbBr_3_ QDs,^9^ this result puts forward the exciton-polaron in CsPbBr_3_ QDs as a mix between a large and a small polaron.

Our study provides the first structural evidence that resonant excitation in the single exciton limit leads to the formation of exciton-polarons in CsPbBr_3_ quantum dots. By highlighting the unmatched sensitivity of SFX to small reorganizations of the atomic lattice, such as the strain profile associated with a single exciton-polaron, this result creates a vast range of opportunities for researchers to investigate the interaction between free charges, excitons, or excitonic complexes and the atomic lattice. Given the femtosecond time resolution of the approach, one can envision monitoring the dynamics of polaron formation and relaxation in reciprocal space by sweeping the pump–probe time delay. In future experiments, the serial approach can also be used to classify snapshots by the size and shape of the QDs, creating a subset of frames that can be studied quantitatively.^39,40^ For a perfectly homogeneous ensemble, the 3D diffraction pattern is directly related to the Fourier transform of the electron density in the QDs.^25^ In that way, the deformation field accompanying a polaron can be unambiguously identified,^41^ a major step beyond what ensemble-averaging powder diffraction approaches can achieve. Further studies on the lattice response for hybrid perovskite QDs, noncubic particle morphologies, alternate surface capping ligands, and cryogenic temperatures would complete the picture of this rich physics.

## Methods

### Pump–Probe Aerosol Serial Femtosecond Crystallography

Measurements were performed in the upstream interaction chamber of the Single Particles Biomolecules and Clusters/Serial Femtosecond Crystallography (SPB/SFX) instrument at the European XFEL. 13.6 keV X-ray photons were focused to a 200 nm spot using Kirkpatrick–Baez mirrors. The Adaptive Gain Integrating Pixel Detector (AGIPD) was placed 122.4 mm downstream of the interaction region to collect the diffraction from the aerosolized particles. Diffraction patterns were collected at a rate of 3520 frames/s, with approximately 1% of the frames containing measurable diffraction from nanocrystals.

In order to deliver the samples to the X-ray beam, nanocrystals were aerosolized using electrospray ionization and then transported and focused to the X-ray beam using an aerodynamic lens stack.^28^ Electrospray ionization requires a conductive solvent in order to obtain the Taylor cone necessary for the formation of small droplets. To achieve the aerosolization of the CsPbBr_3_ NCs that do not survive in polar solvents, they were dispersed in toluene, and 2% ionic liquid (trihexyltetradecylphosphonium bis­(trifluoromethylsulfonyl)­amide) was added to make the solvent conductive. Prior tests showed that the QDs survived in this environment for at least a few hours before the NCs dissolved, as evidenced by the formation of lead bromide precipitates and the dispersion not fluorescing.

### Data Reduction and Recalibration

Peak finding to detect patterns with crystalline diffraction was performed with the OnDA implementation^42^ of the *peakfinder8* algorithm.^43^ The crystal hits were indexed using a modified version of the *SPIND* algorithm.^44^ Instead of pregenerating a large reference table that consists of all possible pairs of ideal Bragg peaks as in the original *SPIND* implementation, we leveraged the fact that the |**q**| of Bragg peaks in reciprocal space are orientation-independent and generated the reference pairs on the fly. Multiple crystal indexing was done by removing the previously indexed peaks in each step and attempting indexing again. To increase the indexing success rate, we adopted an adaptive criterion for accepting a set of indexed peaks as a crystal. In the first round, we set the criterion to find at least four peaks that fit a single lattice. In the second round, for patterns where we could not find any crystal, we lowered the criterion to 3 peaks.

The orientations of the indexed crystals, denoted as a matrix Ω, were further refined by explicitly modeling the cross shape of the Bragg peaks, resulting from the cubic shape of the NCs. Specifically, we minimized the following target function against Ω

1 $$\sum_{q,s\in c\mathrm{rystal}}s\cdot \exp\left(-\frac{1}{2W^{2}}\left(|\Omega q{|}^{2}-r\cdot \max_{i\in \left\{x,\mathrm{yz}\right\}}{\left(\Omega q\right)}_{i}^{2}\right)\right)$$

where *s* is the integrated peak intensity. The hyperparameters were the peak width *W* = 4.6 voxel and the bias ratio *r* = 0.3.

Since we were interested in both the strong peak intensities as well as the weak tails of the peaks, the AGIPD was operated in gain-switching mode. The patterns containing peaks were recalibrated using a custom procedure that used the data from the in-pixel constant current source^45^ to better calibrate pixels in the regions where the gain signal of the AGIPD indicated the pixel was close to, or had crossed, the gain-switching threshold.

### 3D Intensity Generation

The orientations from indexing were used to rotate the Ewald sphere for the entire frame and merge it into a 3D reciprocal space. To account for the effects of beam fluence, sample crystal size, and partiality of Bragg peaks, the indexed crystals of each frame were independently rescaled using different rescaling factors. The rescaling process involved first merging the intensities from all of the indexed crystals together to create a 3D intensity reference without any rescaling. Then, for each crystal, the corresponding Ewald sphere cut of the reference intensity was extracted from the 3D intensity map, and a rescaling factor for that crystal was determined by calculating the ratio between the mean intensity of the brightest 10 pixels in the reference cut and the corresponding crystal’s mean intensity.

To refine the intensity reconstruction, we implemented a process to remove crystals that deviated significantly from their corresponding merged intensity slice. For each indexed crystal, following normalization to the brightest pixel, a selected set of pixels of the pattern were compared to the merged intensities. These pixels were within a radius of 4 pixels from the expected Bragg peaks, which had expected intensities greater than 0.01 times the mean intensity. The scalar product of these pixels with the corresponding slice of the 3D merged intensity was used as a metric to reject indexed crystals, which were too dissimilar.

Before merging the frames into reciprocal space, the pixels in the neighborhood of peaks in the pattern, but not indexed into the lattice, were excluded. This significantly reduced the artifacts in the merged volume from multicrystal diffraction patterns. Background subtraction was carried out in two sequential steps for the indexed crystal patterns. First, the radial average for each pattern was calculated, excluding outlier values from the vicinity of Bragg peaks, generating a one-dimensional (1D) array depicting the background variation with respect to |**q**| for each frame. Following this, singular value decomposition (SVD) was applied to these 1D feature arrays, with the first three components employed to reconstruct the background.

### DFT Calculation Details

Theoretical calculations were conducted at the density functional theory (DFT) level using the semilocal PBE exchange-correlation functional,^46^ as implemented in the CP2K 2024.1 package.^47^ A double-ζ basis set, augmented with polarization functions, was employed alongside effective core potentials for all atom types.^48^ A standardized protocol was followed: first, a manually selected initial structure was relaxed to its ground state, defined by a spin multiplicity of 1. The resulting optimized ground-state structure was then used as the starting point for relaxing the excited-state structure, defined by a spin multiplicity of 3. For both states, the electron density was subsequently calculated via single-point calculations and exported as cube files that contain the atomic coordinates and electron density on a 3D grid. These data were then used to determine the diffraction patterns for both the ground and excited states.

The analysis was carried out on a charge-neutral nanocrystal (NC) model with stoichiometry Cs_200_Pb_125_Br_450_. The NC adopts an approximately cubic shape, consisting of 5 cubic unit cells extending in each direction, as illustrated in Figure 4a. Structurally, this composition results in an inner framework of 5 × 5 × 5 Pb atoms, surrounded by an outer framework of 6 × 6 × 6 Cs atoms. To achieve a total of 200 Cs atoms, 16 vacancies were introduced into the outer Cs layer. Specifically, all 8 Cs atoms at the vertex positions were removed, and in most cases, the remaining 8 vacancies were positioned along the edges. Different arrangements of these vacancies give rise to distinct model NCs. A comprehensive description of these variations is provided in Supporting Information S6.

### Intensity Calculation for Different Polaron Models

The scattered intensity from a single crystal was calculated by performing a 3D Fourier transform of the electron density of the crystal, represented by point-like atoms weighted by their tabulated scattering factors at 13.6 keV. The simulated intensities for the undistorted crystal were calculated by incoherently averaging the scattered intensities from crystals sampled from a size range between 3 and 15 nm weighted by the γ distribution with a shape parameter of 2.0 and an average size of 5.4 nm. The orthorhombic basis reported in Materials Project ID mp-567629 ^49^ was used to place atoms in the unit cell and the crystal was truncated along the ⟨100⟩ directions of the cubic unit cell. The shape of the crystal for each size was calculated by using a superellipsoid envelope with an exponent of 5.0 in order to partly round the corners and more closely match the observed truncation rods in the measured 3D intensities.

For the orthorhombic relaxation model, the displacement of the atoms from the cubic lattice was parametrized by the linear parameter *t* such that *t* = 0 represented the cubic lattice and *t* = 1 represented the tabulated orthorhombic structure. The distortion from the perfect orthorhombic structure for atom *i* is then

2 $$\delta_{i}=tr_{o,i}+\left(1-t\right)r_{c,i}$$

where **r**_o|c,*i*_ represents the position of atom *i* in the orthorhombic or cubic lattice, respectively. Supporting Information S4 shows an illustration of this distortion in real space. The results shown in Figure 3c were obtained by varying the *t* parameter from 0.8 for the unit cell next to the center and decaying to 1 with the square of the distance of the unit cell to the origin. This model assumes the octahedral rotation decreases due to the polaron. Assuming an increase of the octahedral rotation close to the center of the QD results in the opposite sign for intensity changes compared to that displayed in Figure 3c.

For the random hole localization model, the normalized hole positions, **r̃**_*h*_, were randomly generated using the following algorithm

3 $${\mathrm{r̃}}_{h}≔\left(U\left(-1,1\right),U\left(-1,1\right),U\left(-1,1\right)\right)$$

4 $${\mathrm{r̃}}_{h}≔{\mathrm{r̃}}_{h}\frac{{∥{\mathrm{r̃}}_{h}∥}_{5}^{0.1}}{{∥{\mathrm{r̃}}_{h}∥}_{5}}$$

where U(−1,1) is a uniform random number between −1 and 1 and ∥·∥_5_ refers to the *l* ^5^-norm of a vector. The actual position was then estimated by scaling this vector by the size of the sampled NC.

For simplicity, the electric field from the delocalized electron charge was calculated from a sphere model

5 $$E\left(r\right)\propto \frac{r}{{\left(s/2\right)}^{3}}$$

where *s* is the size of the sampled NC.

## Supplementary Material



## Notes

Additional modeling and simulation details, including data collection and validation statistics (PDF)
