# Supplementary material for: Direct Observation of the Exciton-Polaron in Single CsPbBr3 Quantum Dots
Source: ACS Nano. 2025 Aug 2;19(31):28372–82. doi: 10.1021/acsnano.5c06716 (PMC12356124; doi:10.1021/acsnano.5c06716)
Supplement: Supplementary file 1 [file nn5c06716_si_001.pdf]

Supporting Information: Direct observation of the exciton polaron in single $CsPbBr_3$ quantum dots

Zhou Shen,[1] Margarita Samoli,[2] Onur Erdem,[2] Johan Bielecki,[3] Amit K. Samanta,[4] Juncheng E,[3] Armando D. Estillore,[4] Chan Kim,[3] Yoonhee Kim,[3] Jayanath Koliyadu,[3] Romain Letrun,[3] Federico Locardi,[5,2] Jannik Lübke,[4] Abhishek Mall,[1] Diogo V. M. Melo,[3] Grant Mills,[3] Safi Rafie-Zinedine,[3] Adam Round,[3] Tokushi Sato,[3] Raphael de Wijn,[3] Tamme Wollweber,[1,6] Lena Worbs,[4] Yulong Zhuang,[1] Adrian P. Mancuso,[3,7] Richard Bean,[3] Henry N. Chapman,[4,6,8] Jochen Küpper,[4,6,8] Ivan Infante,[9,10] Holger Lange,[6,11] Zeger Hens,[2,12,*] and Kartik Ayyer[1,6,†]

[1] *Max Planck Institute for the Structure and Dynamics of Matter, 22761 Hamburg, Germany*

[2] *Physics and Chemistry of Nanostructures, Ghent University, Gent 9000, Belgium*

[3] *European XFEL, 22869 Schenefeld, Germany*

[4] *Center for Free-Electron Laser Science CFEL, Deutsches Elektronen-Synchrotron DESY, 22607 Hamburg, Germany*

[5] *Dipartimento di Chimica e Chimica Industriale, Università degli Studi di Genova, 16146 Genova, Italy*

[6] *The Hamburg Center for Ultrafast Imaging, 22761 Hamburg, Germany*

[7] *Department of Chemistry and Physics, La Trobe Institute for Molecular Science, La Trobe University, Melbourne, VIC 3086, Australia*

[8] *Department of Physics, Universität Hamburg, 22761 Hamburg, Germany*

[9] *BCMaterials, Basque Center for Materials, Applications, and Nanostructures, UPV/EHU Science Park, Leioa, 48940 Spain*

[10] *Ikerbasque Basque Foundation for Science, Bilbao 48009, Spain*

[11] *University of Potsdam, Institute of Physics and Astronomy, 14476 Potsdam, Germany*

[12] *NoLIMITS Center For Non-Linear Microscopy and Spectroscopy, Ghent University, Gent 9000, Belgium*

* zeger.hens@ugent.be
† kartik.ayyer@mpsd.mpg.de

S1. DATA COLLECTION STATISTICS

Metric	Dark	Light	Total
Total frames	17 582 224	17 482 325	35 064 549
Hit frames	441 071	439 302	880 373
Hit rate	2.51%	2.51%	2.51%
Frames with peaks	39964	37218	77182
Peak hit rate	0.227%	0.213%	0.220%
Indexed frames	10106	9997	20103
Indexed crystals	15842	15705	31547
Indexing rate	39.6%	42.2%	40.8%
Indexed peaks	79855	79805	159660

TABLE S1. Data collection statistics for the dark (umpumped) and light (3 ps after optical excitation) serial femtosecond crystallography datasets.

S2. AVERAGE QUANTUM-DOT OCCUPATION

A. Absorption cross section and laser fluence

We obtained the cross section σ_{exc} at the excitation wavelength from published values of the intrinsic absorption coefficient $\mu_{i,335}$ of $CsPbBr_3$ quantum dots (QDs) at 335 nm [1]:

$$\sigma_{exc} = \mu_{i,exc} \times V_{QD} = \left(\mu_{i,335} \frac{A_{exc}}{A_{335}} \right) \times V_{QD}$$

Here, V_{QD} is the QD volume. For the given sample, we thus obtained:

$$\sigma_{477} = 6.16\ 10^{-15}\ \mathrm{cm}^2$$

We subsequently set the laser fluence J such that the photon flux ϕ_{477} was equal to $1/\sigma_{477}$. Hence:

$$J = 67.6\ \mu\mathrm{J} \cdot \mathrm{cm}^{-2}$$

B. Average quantum-dot occupation

$CsPbBr_3$ quantum dots (QDs) feature a mixed confinement regime, with a 2-fold degenerate electron state and a localized hole state. For the QDs studied here, formation of one electron-hole pair reduces the absorption cross-section for resonant excitation to $\approx 40\%$ of the initial value [2]. Furthermore, the complete occupation of the electron states blocks additional absorption to form 3 electron-hole pairs. We therefore estimate the fraction of QDs having (P_0) no, (P_1) one and (P_2) two electron hole pairs using the following set of dynamic equations:

$$\frac{dP_0}{dt} = -\gamma_0 P_0$$
$$\frac{dP_1}{dt} = \gamma_0 P_0 - \gamma_1 P_1$$
$$\frac{dP_2}{dt} = \gamma_1 P_1$$

We thus obtain:

$$P_0 = e^{-\gamma_0 t}$$
$$P_1 = \frac{\gamma_0}{\gamma_0 - \gamma_1}\left(e^{-\gamma_1 t} - e^{-\gamma_0 t}\right)$$
$$P_2 = 1 - P_0 - P_1$$

For the fluence chosen, we have $\gamma_0 t = 1$ and $\gamma_1 t = 0.4$ at the end of each laser pulse. One thus obtains:

$$P_0 = 0.368$$
$$P_1 = 0.504$$
$$P_2 = 0.128$$

Since the 3 ps delay between the optical pump and the X-ray probe is shorter than the lifetime of two electron-hole pairs in a single QD [2], we assume that the X-rays probe QDs with the above occupation probability. Assuming that two electron-hole pairs yield double the distortion as one electron-hole pair, we thus obtain that on average, $0.504 + 2 \times 0.128 = 0.760$ electron/hole pairs are probed per pulse.

S3. FRACTION INTENSITY DIFFERENCES

Figure 3b of the main text shows the absolute intensity differences between the pumped and unpumped (light and dark) datasets in the neighborhood of the 200, 400, 110 and 111 peaks. The fractional differences $(I_{\text{light}} - I_{\text{dark}})/I_{\text{dark}}$ are shown for the same regions in Fig. S1a. Since the intensities are negligible far from the peak, Fig. S1b shows the differences only for voxels where the I_{dark} is greater than 5% of the maximum value.

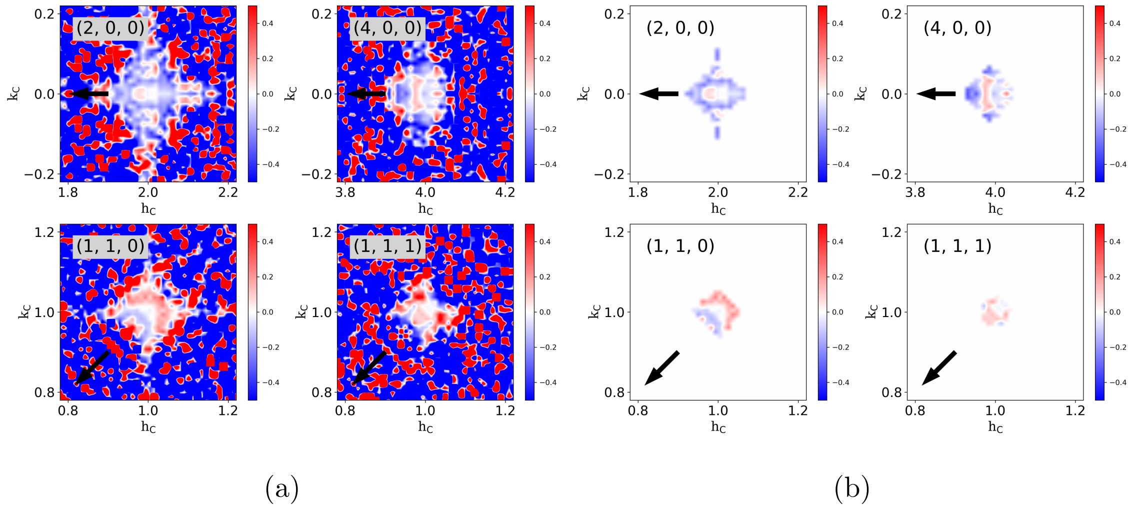

FIG. S1. Fractional intensity difference maps (a) For all voxels in the neighborhood of the 200, 400, 110 and 100 peaks, which are the same as in Fig. 3b of the main text. (b) Same plot, but with the low intensity regions masked.

S4. ORTHORHOMBIC RELAXATION MODEL

The Jahn-Teller (J-T) distortion associated with the orthorhombic structure is associated with the rotation of the Br octahedra to break the electronic degeneracy. One possible polaronic lattice distortion model is to use this low-energy octahedral rotation mode. In this picture, the exciton causes the degree of octahedral tilt to vary as a function of distance from the centre of the polaron. In Fig. S2b, the orthorhombic J-T distortion is relaxed near the centre and approaches the equilibrium value further away. The two extreme cases are shown in Fig. S2a and c.

The effect of such a variable tilt distortion is shown in Fig. 3c of the main text, where no peak shifts are observed, but only changes in the total intensities. Note that the effect is strongly exaggerated for the purposes of illustration in Fig. S2b.

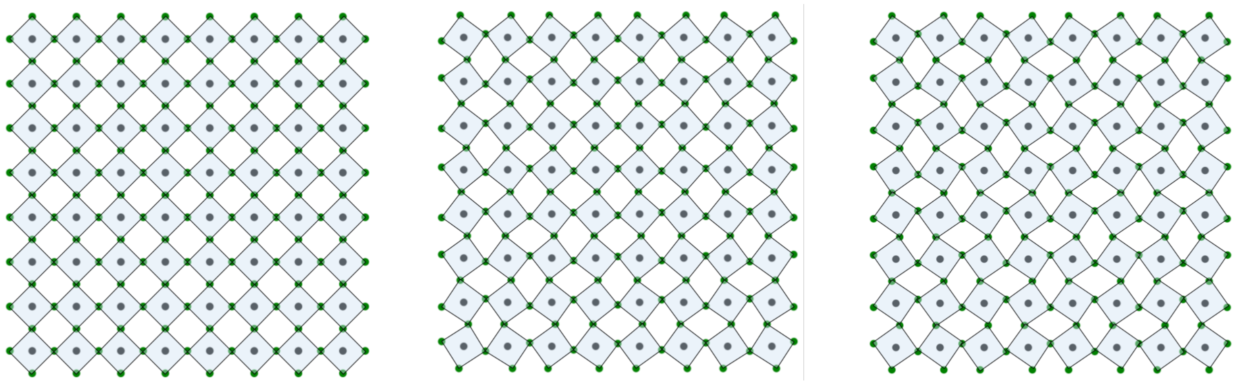

(a) (b) (c)

FIG. S2. Three different octahedral rotation models (a) No rotation, equivalent to the cubic structure. (b) Variable rotation, with smaller tilts near the centre of the particle. (c) Maximal rotation corresponding to the equilibrium orthorhombic structure.

S5. HALF-DATASET VALIDATION

A. Intensity difference maps

In order to validate the statistical significance of the intensity difference maps in Fig. 3 of the main text, we perform half-dataset tests analogous tot he gold-standard Fourier shell correlation (FSC) measurements in cryo-electron microscopy. Each of the dark and light datasets were split into two half-datasets (even and odd frames), which were independently averaged and merged. The differences of the even and odd merges were independently calculated and are shown in Fig. S3a and b. For reference, Fig. 3b of the main text is reproduced in Fig. S3c. One can see that the peak shifts are reproduced in the half-dataset merges, albeit with reduced signal-to-noise. Many of the other, higher order, features are not as reproducible and we do not use them in formulating the model described in the main text.

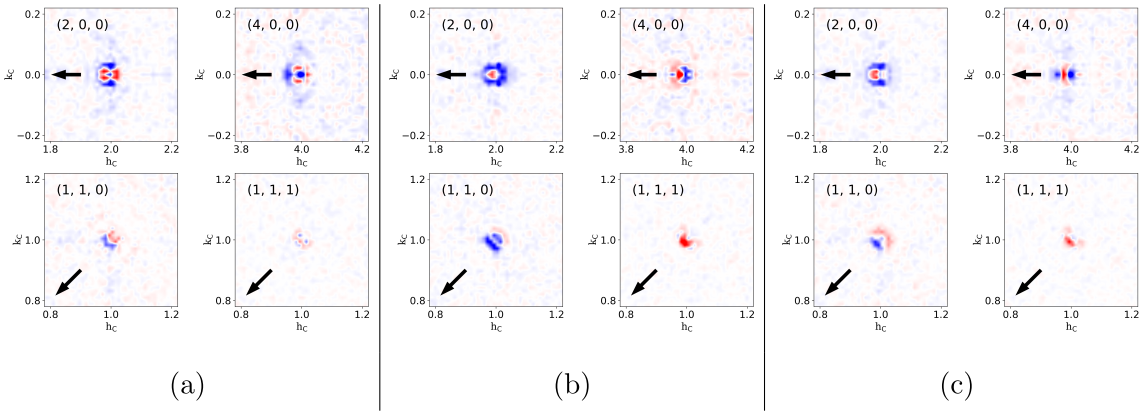

FIG. S3. Half-dataset intensity differences in the neighborhood of the 200, 400, 110 and 111 reflections. (a) For the odd frames in the dark and light datasets (b) Even frames (c) All frames (same as Fig. 3b of the main text).

B. Integrated Bragg reflections

While the modelling has focussed on changes in the 3D peak profile of the Bragg reflections, one must also check whether the integrated Bragg intensities change upon optical excitation. This would reflect a change in the average unit cell structure, and would also be reflected int eh virtual powder pattern of Fig. 2b of the main text. In order to check

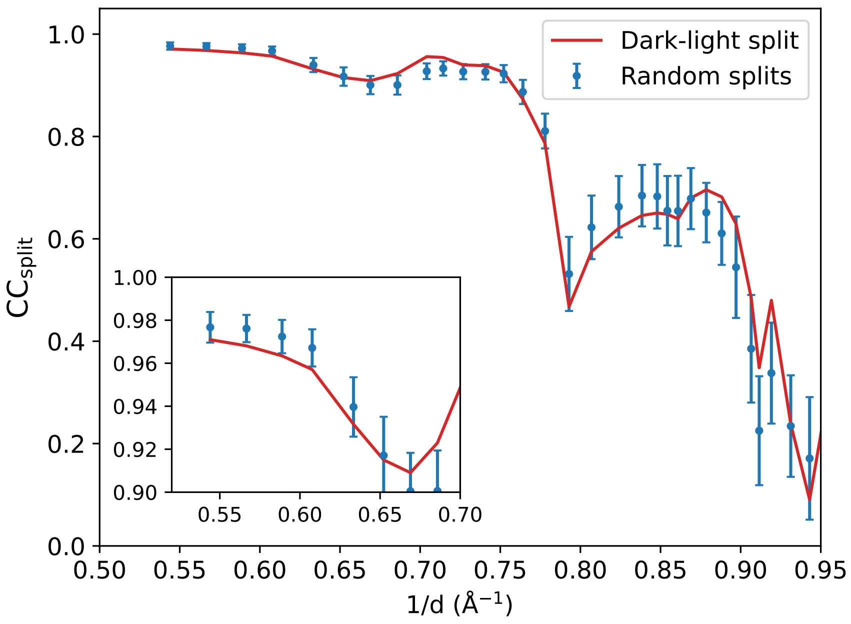

FIG. S4. Integrated intensity correlation coefficient (CC) as a function of momentum transfer. The blue line with error bars depicts the half-dataset CC for random splits combining both pumped (light) and unpumped (dark) datasets. The red line shows the CC between the dark and light datasets, and it is apparent that this is not significantly lower than a half-dataset split.

the statistical significance of the change in integrated intensities, we perform the following test. The whole dataset (pumped and unpumped together) are split into half datasets 50 times randomly. In each case, the integrated *hkl* intensities are calculated and their Pearson correlation coefficient is calculated in resolution bins. This is analogous to the $CC_{1/2}$ metric commonly used in SFX. Due to random fluctutations and noise, the $CC_{1/2}$ is not unity, but varies with resolution as shown in Fig. S4 (error bars depict one standard deviation). If optical excitation notably modified the integrated intensities, the $CC_{1/2}$ between the dark and light datasets should be significantly lower than a random split. As one can see from the red line in Fig. S4, this is not the case, resulting in our conclusion that we do not observe any significant change to the integrated intensities, and the high sensitivity of 3D intensity difference maps are necessary.

S6. DENSITY FUNCTIONAL THEORY ANALYSIS OF LATTICE DEFORMATION IN $CsPbBr_3$ QUANTUM DOTS

A. Computational approach

1. *General methodology*

To analyze the formation of a polaron in $CsPbBr_3$ QDs, we compared the relaxed QD geometry as obtained for the electronic ground state and the first excited state as predicted by density functional theory (DFT). These geometries were obtained by imposing either a total spin $S = 0$, or a total spin $S = 1$. Clearly, the latter will give the triplet state of the exciton, but the assumption is that the relaxation of the QD geometry will be highly similar for the singlet and triplet exciton. The DFT analysis provides the electron density for the valence electrons, and the (x, y, z) coordinates of the different atoms. Using this information, 3D scattering patterns can be calculated for the ground state and the excited state by Fourier transformation, where the difference in patterns provides a result that can be compared directly with experimentally measured differences in diffraction intensity.

DFT calculations were implemented in CP2K, using the PBE functional. A fixed sequence was followed, in which first a hand-picked structure, see next section, was relaxed in the ground state. The resulting geometry was then used as an input for relaxing the excited state structure, which was defined by setting the spin multiplicity to 3 instead of 1. For both structures, the electron density was subsequently calculated using an energy calculation. The `.xyz` and `.cube` files providing the atomic coordinates and the electron density on a 3D grid were then taken as an input to determine the diffraction pattern for the ground and excited state. All spatial dimensions are expressed in units of the Bohr radius, $a_0 = 0.529$Å.

2. *The* $CsPbBr_3$ *quantum dot models*

The analysis made use of different charge neutral $CsPbBr_3$ QD models with brute formula $Cs_{200}Pb_{125}Br_{450}$. All models were cut as cubes from a bulk $CsPbBr_3$ crystal creating an inner framework of $5 \times 5 \times 5$ Pb atoms, and an outer framework of $6 \times 6 \times 6$ Cs atoms. To attain 200 Cs atoms, 16 vacancies were created in the outer Cs layer. For all models, the 8 Cs atoms from the cube corners were removed. For most other models, 8 additional Cs atoms

were removed in different patterns from the cube edges. In one model, 4 Cs atoms were removed from edges, and 4 Cs atoms from facets.

B. Calculation of diffraction patterns

1. *The diffraction pattern of the valence electrons*

The valence electron density was computed as a volumetric quantity on an equidistant 3D coordinate grid. To obtain the diffraction pattern with sufficient resolution in reciprocal space, the Fourier transform of the electron density was determined by calculating the Fourier integral across a limited region in reciprocal space around a given k-point. A reduction from a 3D to a 2D problem was achieved by first projecting the electron density on a given plane (in practice, the xy, yz and zx planes), after which the intersection of 3D reciprocal space with the corresponding 2D plane was obtained from a Fourier transform of the projected density:

$$F_{val}(q_1, q_2) = \int \rho_{12}(x_1, x_2) e^{i(q_1 x_1 + q_2 x_2)} dx_1 dx_2 \tag{S1}$$

Here, x_1 and x_2 are the coordinates of the relevant coordinate plane, ρ_{12} the projected electron density and (q_1, q_2) the coordinates in the corresponding plane in reciprocal space.

2. *The diffraction pattern of the core electrons*

The contribution of the core electrons to the diffraction pattern was determined by considering each core atom as a delta-point scatterer, such that the diffraction amplitude $F_{core}(\boldsymbol{k})$ could be determined by a direct summation of the relevant phase factors:

$$F_{core}(\boldsymbol{q}) = \sum_i N_i e^{i\boldsymbol{q}\cdot\boldsymbol{r}} \tag{S2}$$

Here, the index i labels all different core atoms, and N_i is the number of core electrons for each atom as used in the DFT calculation. By selecting $\boldsymbol{q}$ vectors using the same grid as for the valence electrons, a mutually compatible diffraction amplitude is obtained.

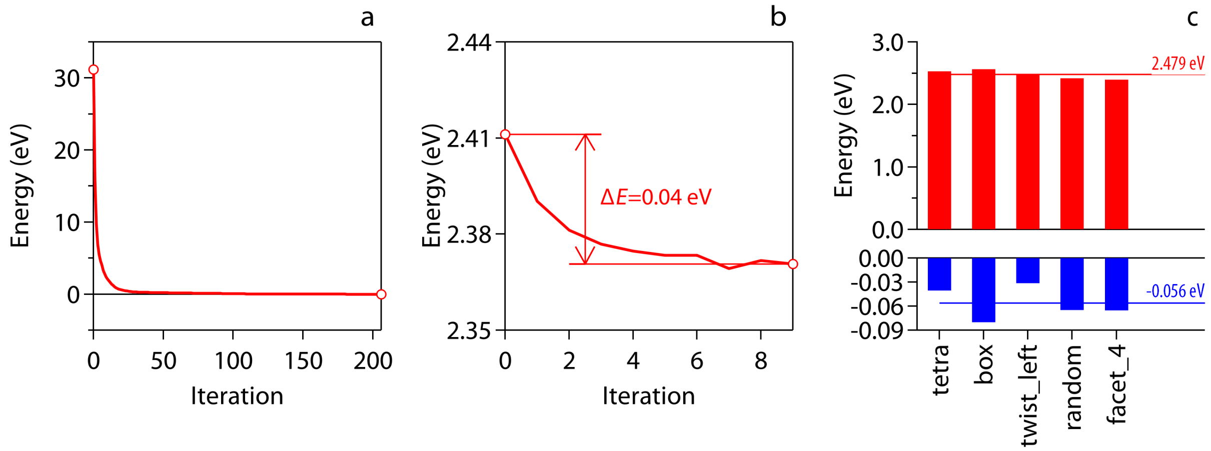

FIG. S5. (a) Geometry optimization through relaxation of the ground state energy for the tetra NC, starting from the hand-picked structure. (b) Geometry optimization through relaxation of the excited-state energy for the tetra NC, starting from the relaxed ground-state structure. For (a) and (b), the energy of the relaxed ground state is taken as the energy reference. (c) Representation of (red) the energy difference between the relaxed excited state and the relaxed ground state and (blue) the relaxation energy for the excited state for the different model NCs. These quantities can be compared to the experimental photon energy of the band-edge emission and the Stokes shift.

3. The diffraction intensity difference

For a given 2D planar slice in reciprocal space, the 2D diffraction difference pattern ΔI_{diff} is determined from $F_{val}(q_1, q_2)$ and $F_{core}(q_1, q_2)$ according to:

$$\Delta I_{diff}(q_1, q_2) = |F_{ES,core}(q_1, q_2) + F_{ES,val}(q_1, q_2)|^2 - |F_{GS,core}(q_1, q_2) + F_{GS,val}(q_1, q_2)|^2 \quad \text{(S3)}$$

C. Model nanocrystal relaxation

1. Energy changes upon relaxation

Figure S5a represents the evolution of the total energy of the tetra NC during the geometry optimization of the ground state, starting from the hand-picked structure. Similar relaxation curves are obtained for all the model NCs analyzed. As shown in Figure S5b, starting from the ground-state structure, the geometry optimization for the excited state leads to a small but systematic energy relaxation. Averaged over the different model NCs,

a relaxation energy of 0.056 eV is obtained, while the energy difference between the relaxed excited state and ground state amounts to 2.479 eV. In principle, the Stokes shift between the band-edge absorption and emission would amount to twice the relaxation energy, while the energy of the band-edge transition in absorption would correspond to the sum of the relaxed energy difference and the relaxation energy.

2. Ground state / excited state atom displacement field

As the core electrons will dominate the diffraction pattern, we first look at the changes in position of the core atoms when comparing the excited state and the ground state. Figure 4b provides, as an example, the colour coded shifts along the x direction of the Cs, Pb and Br atoms. Here, blue means a shift to the right (increasing x) and red means a shift to the left (decreasing x). As can be seen from the scatter plots, the Cs and Pb atoms at the left and the right of the NC feature shifts in line with their position along the x-axis, i.e., an outward displacement. The Br atoms, on the other hand, exhibit a shift opposite to their position along the x-axis, i.e., an inward displacement. A similar pattern emerges along the y and z directions, and appears for the four other NC structures analysed as well.

Figure 4b suggests that shifts along the x direction are mainly determined by the x coordinate of a given atom, and vice versa for the other axis. Figure S6 provides the shifts of the Br position along x, y and z, plotted as a function of the x, y and z coordinate, respectively. Invariably, a linear regression yields a significant correlation for the shift Δx along x, shift Δy along y, and Δz along z, while all other combinations are either uncorrelated, or weakly correlated. Table S2 provides for each regression line the slope, and the error on the slope.

D. Nanocrystal diffraction patterns

1. Diffraction from the core electrons

Figure S7a represents the diffraction pattern obtained for the relaxed ground state of the tetra NC. The pattern reflects the nearly cubic symmetry of the $CsPbBr_3$ QD lattice, featuring the most intense diffraction peaks for the (200), (220), and (400) directions. Note that the intensity at $k = 0$ amounts to 31550^2, i.e., the square of the total number of core electrons. A detailed view of the diffraction pattern along k_x, k_y, or k_z shows that for this

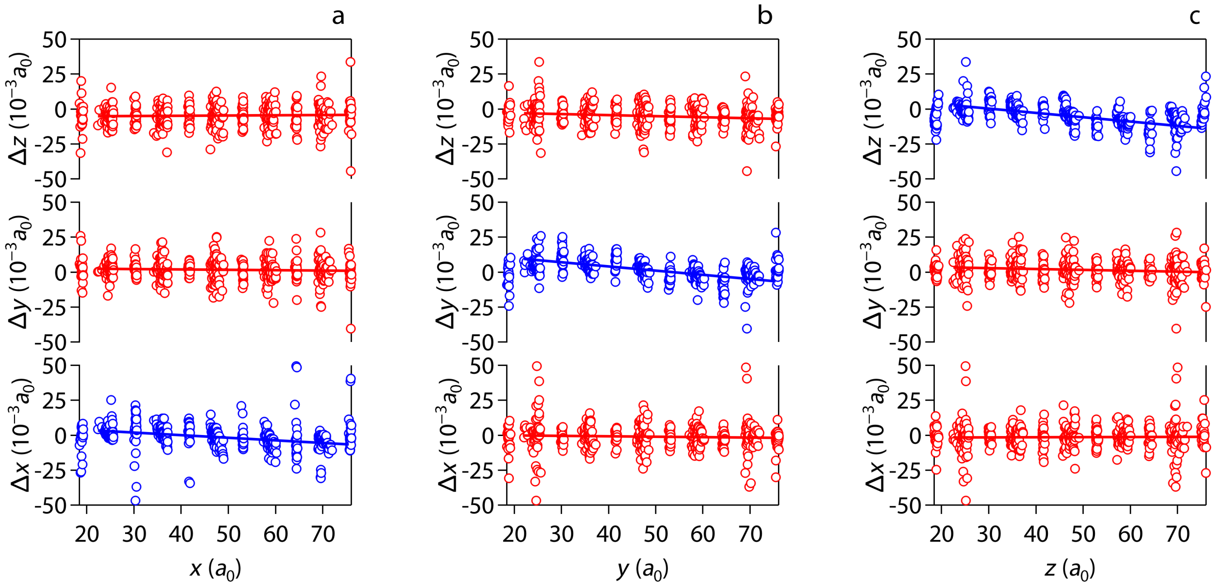

FIG. S6. Shift of Br atoms in the x, y and z direction, plotted as a function of (a-c) the x, y and z coordinate of the Br atoms. All lines represent the result of a linear regression, excluding the Br atoms at the outer surfaces left and right.

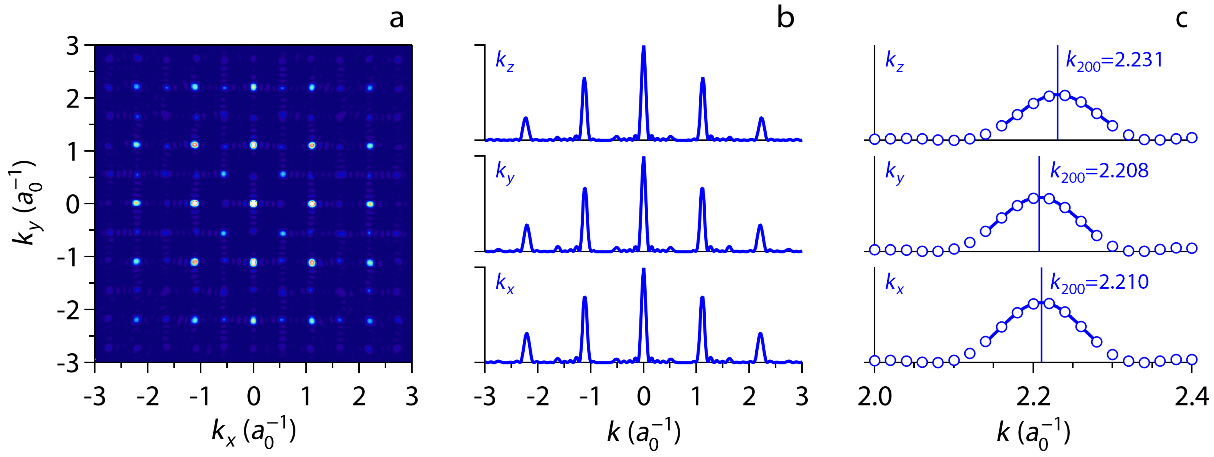

FIG. S7. (a) $k_x - k_y$ slice of the diffraction pattern of the core electrons in the tetra NC. (b) Line intensities along the different direction in reciprocal space as indicated. (c) Zoom on the line intensities around the (400) diffraction peak with a best fit of the central part to a Gaussian, for which the central wavenumber is indicated.

Br Shift/coordinate	x	y	z
Δx	-190±28	-25±25	18±24
Δy	-28±30	-300±22	-75±26
Δz	9±31	-66±27	-311±21
Cs shift/coordinate	x	y	z
Δx	325±25	8±34	-12±34
Δy	-9±71	905±31	52±70
Δz	-16±74	73±71	920±31
Pb shift/coordinate	x	y	z
Δx	386±48	43±45	-20±36
Δy	74±74	302±50	106±35
Δz	13±60	137±36	275±54

TABLE S2. Relative positional shift in a given direction (rows) for atoms along a given direction (columns). Shifts give the slope of linear fits to the atomic displacement, as shown in Fig. S6 for Br in parts per million of the interatomic distance.

NC, the (400) peak attains a maximum at a slightly larger wavenumber along k_z than along k_x and k_y.

The intensity difference between the even 200 and 400 peaks and the odd 100 and 300 peaks results from the phase of the contributions of the different atoms to F_{core}. For even order peaks, all atoms within the unit cell—when perfectly positioned on the lattice points—contribute in phase. Hence, F_{core} is the sum of the scattering from one Pb, one Cs, and three Br atoms per unit cell. For odd order peaks, there is a phase shift of π between the scattering from the Cs and Br atoms at the edge of the unit cell, and the Pb and two Br atoms in the center of the unit cell considered along the (100) direction. Hence, F_{core} is the difference of the contribution of one Pb and one Br, and one Cs atom per unit cell.

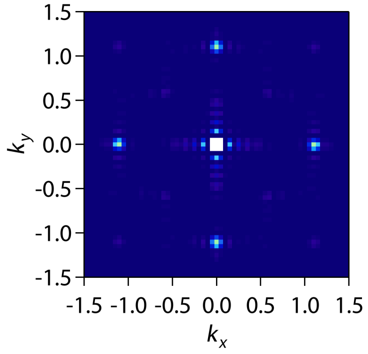

FIG. S8. Low resolution $k_x - k_y$ slice of the diffraction pattern of the valence electrons in the tetra NC.

2. *Diffraction from the valence electrons*

Figure S8 represents a slice of the diffraction pattern obtained from the density of the valence electrons calculated at low resolution. Apart from $k = 0$ diffraction, mostly the (200) diffraction peak is visible. The intensity at the center of the k=0 peak amounts to 29702500=5450^2, i.e., the square of the number of valence electrons. Overall, the contribution of the electron density distribution to the diffraction is far smaller than that of the core electrons. In line with the real space electron density, the (200) diffraction peak is dominant, but far less intense than the (200) diffraction from the core electrons.

E. Excited State / Ground State Diffraction difference patterns

1. *Detailed analysis of the (200) diffraction*

To develop an understanding as to how the atomic shifts lead to a difference between the diffraction pattern of the excited state and the ground state, Figure S9 represents the simulated diffraction difference for the (200) peak, related to only Cs, only Pb or only the Br atoms. In line with the concomitant outward shift of Cs and Pb – which essentially increases the lattice parameter for the Cs and Pb sublattice – one sees that the diffraction related to Cs and Pb becomes more intense for smaller wavenumbers, and loses intensity for larger wavenumbers. The diffraction related to Br exhibits the opposite behavior, losing intensity at small wavenumbers and gaining intensity at large wavenumbers. Note that the shifts are not always entirely oriented towards the center of reciprocal space, an observation

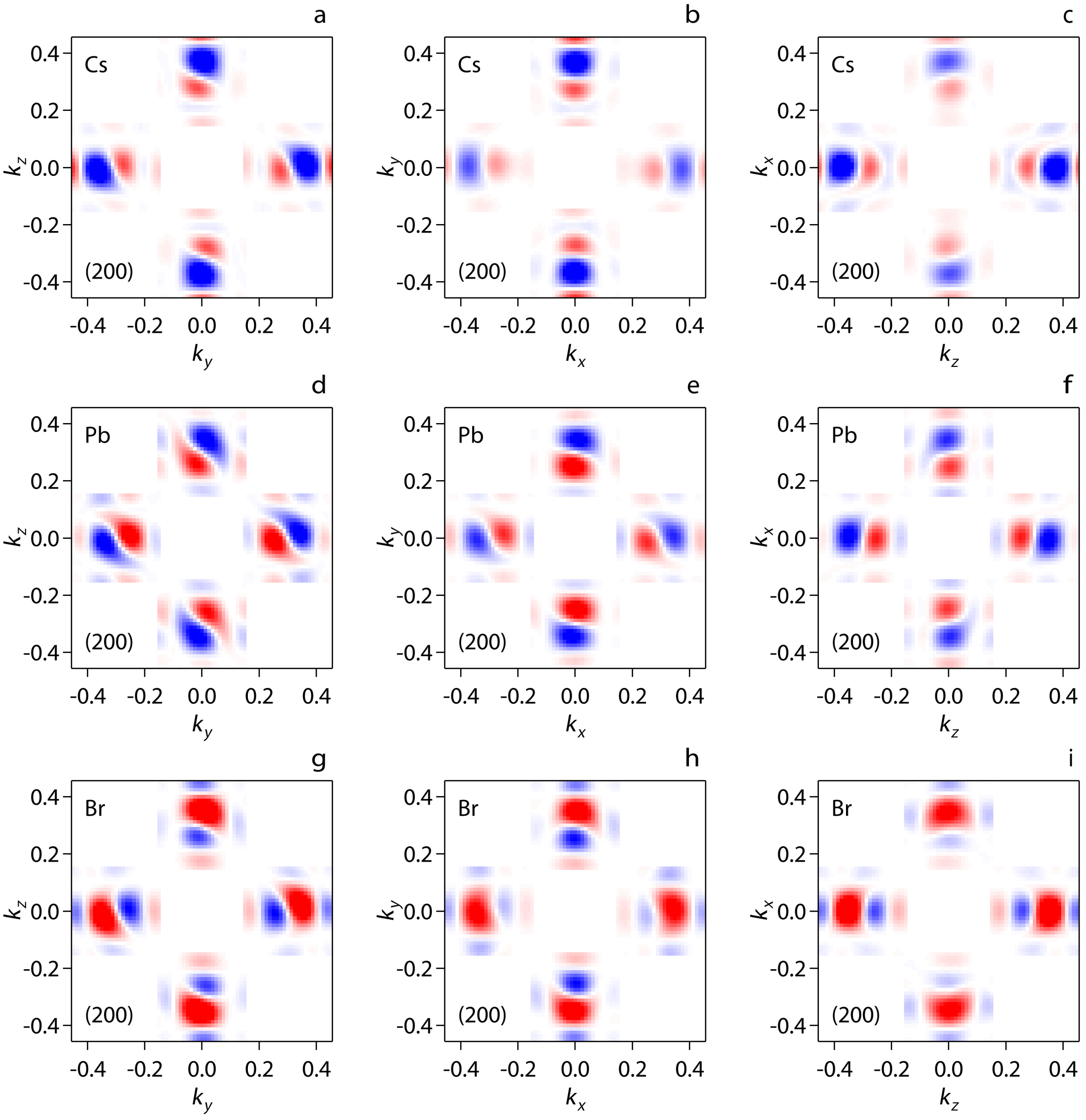

FIG. S9. Single atom diffraction intensity difference simulated for (a) Cs-only, (b) Pb-only and (c) Br-only diffraction. Image plots from left to right represent different slices perpendicular to the 3 main axes in reciprocal space. Red colors represent an increased diffraction intensity in the excited state, blue colors a reduced diffraction intensity. Note that the (200) peaks have been shifted in reciprocal space from a center position at $k = 1.1$ to $k = 0.3$ to enhance the clarity of the patterns.

suggesting that some cross-correlation exists between, e.g, shifts Δz along the y direction. For Br, such a secondary correlation can be deduced from Table S2.

Figure S10a-b represent the total intensity of the (020) diffraction peak in the ground state and the excited state in the $k_x - k_y$ plane. As can be seen, both diffraction features

are highly similar. Even so, the intensity difference shows a systematic variation with an increased intensity at smaller wavenumbers and a decreased intensity at larger wavenumbers after photo-excitation. Note that the intensity difference peaks at about 0.2% of the peak intensity. Figure S10d-f represents the intensity difference map of the (200) peak, counting all contributions from the core and the valence electrons. In line with the line section shown in Figure S10c, one sees that, on the whole, the diffraction intensity increases at smaller wavenumbers, while the diffraction intensity decreases at larger wavenumbers. Referring to Figure S9, this implies that the changes to the diffraction pattern are dominated by the in-phase contributions from the shifts of the Cs and Pb atoms, and not the Br atoms. This result is not unexpected. For the (200) diffraction, the atoms at all lattice positions contribute to the diffraction patterns with the same phase. Hence, notwithstanding slight shifts relative to the lattice position, the diffraction intensity will be the sum of the intensity of the diffraction by the different atoms, such that a change in intensity will directly reflect changes in the atomic position. Since Cs and Pb represent 124 core electrons, while three Br atoms account for only 84, the overall intensity difference is dominated by the shift of the former atoms, not the latter.

2. *Comparing different $CsPbBr_3$ model QDs*

The symmetry of the model QD is affected by the arrangement of the Cs vacancies at the surface. In the case of the box NC, for example, the vacancies are organized such that the x-axis is reduced to a C2 symmetry axis. Opposite to the case of the tetra NC, this arrangement implies that the atom displacement cannot be described as a simple displacement towards or away from the center. Figures S11 and S12 show the different slices through the 200 diffraction intensity differences, calculated for Cs, Pb and Br separately, and considering the total diffraction, for the case of the Facet4 and the Box NC. As can be seen, the Facet4 NC exhibits a diffraction intensity difference highly similar to the tetra NC, with features pointing towards the center of reciprocal space. The Box NC, on the other hand, features more complex diffraction intensity differences that reflect more involved atom displacement fields. On the other hand, the atom-selective patterns still highlight that the displacement of Br is opposite to the displacement of Cs and Pb. This point is a recurring feature of all the model NCs analyzed.

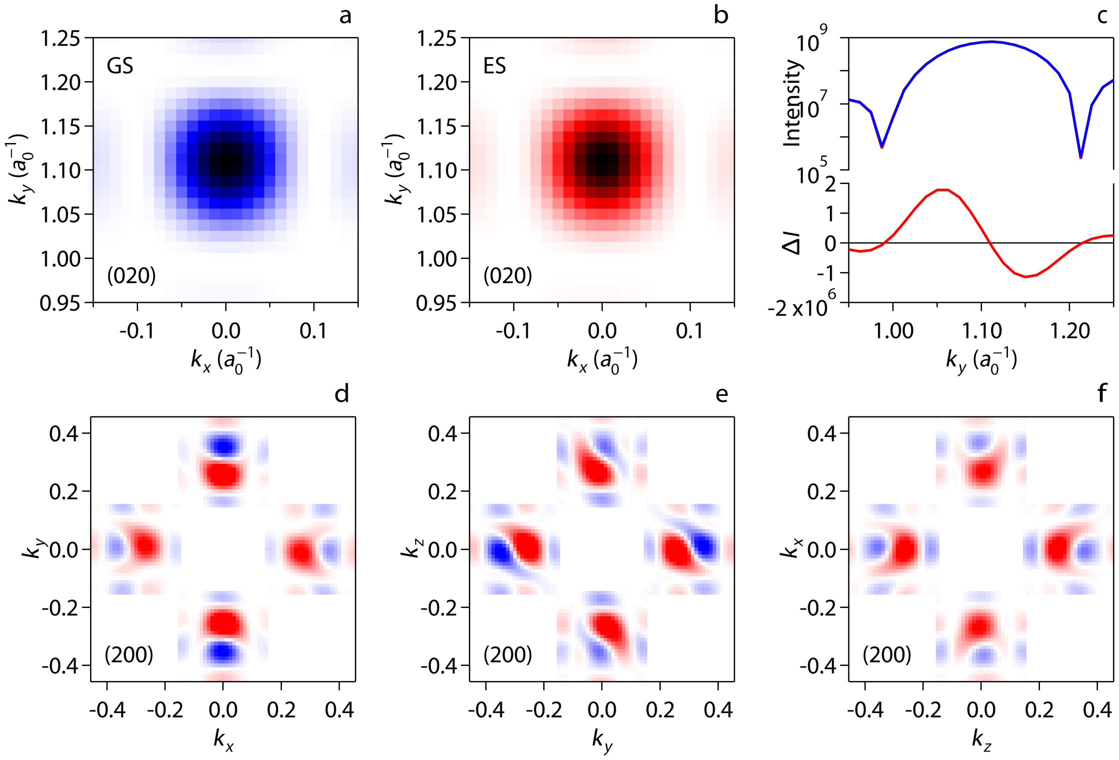

FIG. S10. (a) Slice of the 020 diffraction peak through the $k_x - k_y$ plane for the relaxed ground-state of the tetra NC. (b) The same for the excited state. (c) (top) Diffraction intensity and (bottom) intensity difference of the (020) diffraction peak along the k_y axis. The intensity profiles of the ground and excited states nearly overlap. (d-f) Diffraction intensity difference maps of the (200) peak for intersections with (d) the $k_x - k_y$ plane, (e) the $k_y - k_z$ plane and (f) the $k_z - k_x$ plane.

S7. RESTORING FORCE TUNING

In the Random Hole Localization Model, the lattice displacements are assumed to be in the direction of the local electric field, with the negative Br ions moving opposite to the field. The magnitude of the equilibrium displacement, $\vec{d}$, for each of the atom types will depend on the strength of the restoring force, the effective charge on the atom and local electric field, such that

$$k\vec{d} = q\vec{E} \tag{S4}$$

where k is the *spring constant* of the local harmonic potential.

Assuming a spherically symmetric harmonic potential in the near neighbourhood of the equilibrium position, these restoring strengths can be reduced to a single number for each

atomic species.

For the special case of the centrally localized hole and a delocalized electron, we sampled these three parameters keeping the total squared magnitude constant, i.e. $(k_{\mathrm{Cs}}^2 + k_{\mathrm{Pb}}^2 + k_{\mathrm{Br}}^2)$. For each sampled condition, the differential diffraction was calculated and the peak shift magnitudes for the 200 and 110 peaks were compared.

As one can see in Fig. S13, no choice of relative restoring strength, k, values generate an outward peak shift for the (110) peak. Thus, a centrally localized hole charge cannot account for the observed (110) shift.

For the (200) peak, a relatively weak Br restoring force is required to produce an outward shift. However, this effect reverses for outwardly biased hole positions, wherein a weak Br k-value produces an inward shift. This is also true for the (400) peak and reproduces the final result shown in Fig. 5c of the main text.

[1] J. Maes, L. Balcaen, E. Drijvers, Q. Zhao, J. De Roo, A. Vantomme, F. Vanhaecke, P. Geiregat, and Z. Hens, J. Phys. Chem. Lett. **9**, 3093 (2018).

[2] P. Geiregat, O. Erdem, M. Samoli, K. Chen, J. M. Hodgkiss, and Z. Hens, ACS Nano **18**, 17794 (2024), pMID: 38913946, https://doi.org/10.1021/acsnano.4c03441.

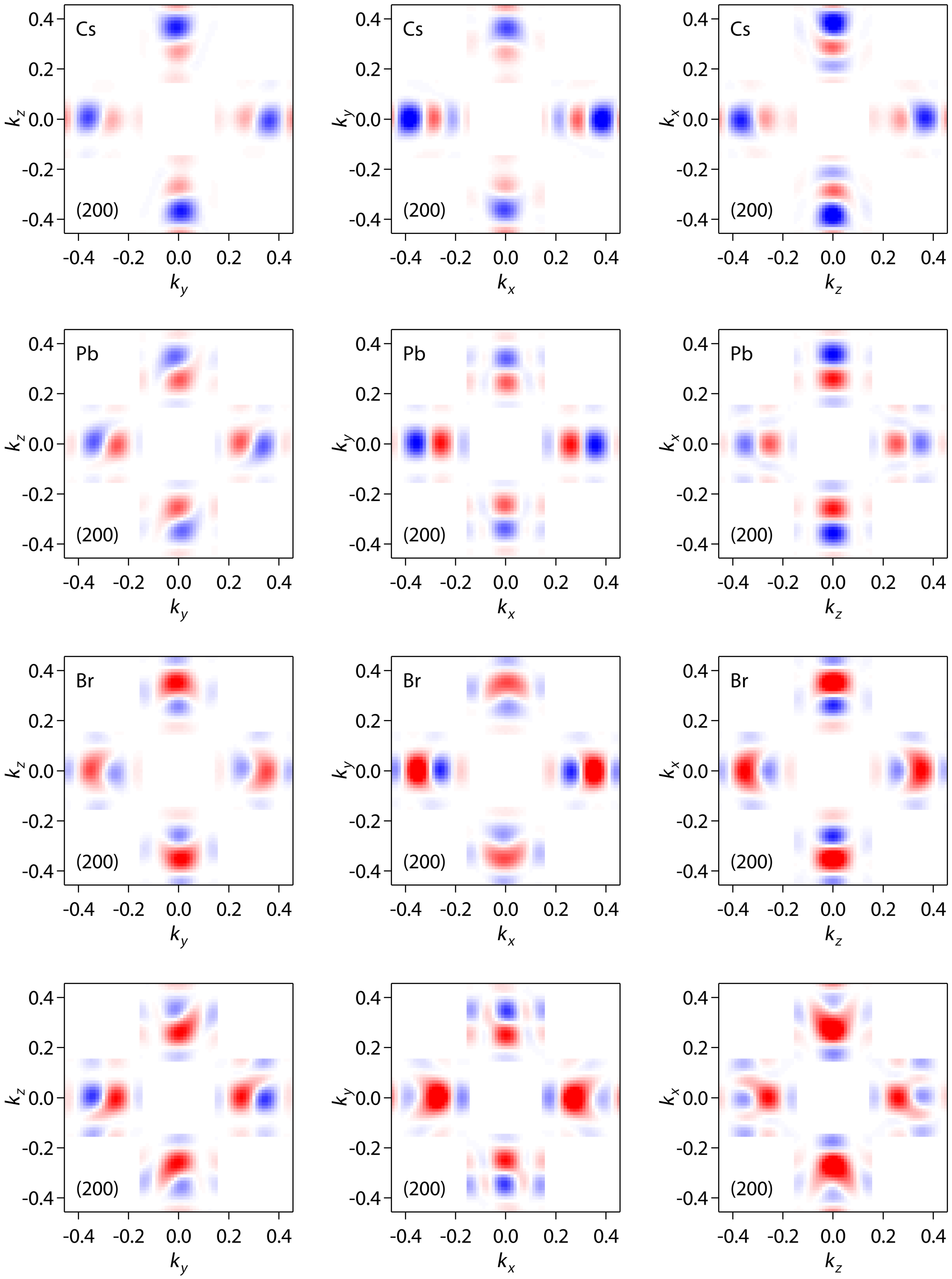

FIG. S11. (3 top rows) Atom-selective diffraction intensity difference maps around the (200) peak and (bottom row) Total diffraction intensity difference around the (200) peak for the Facet4 QD.

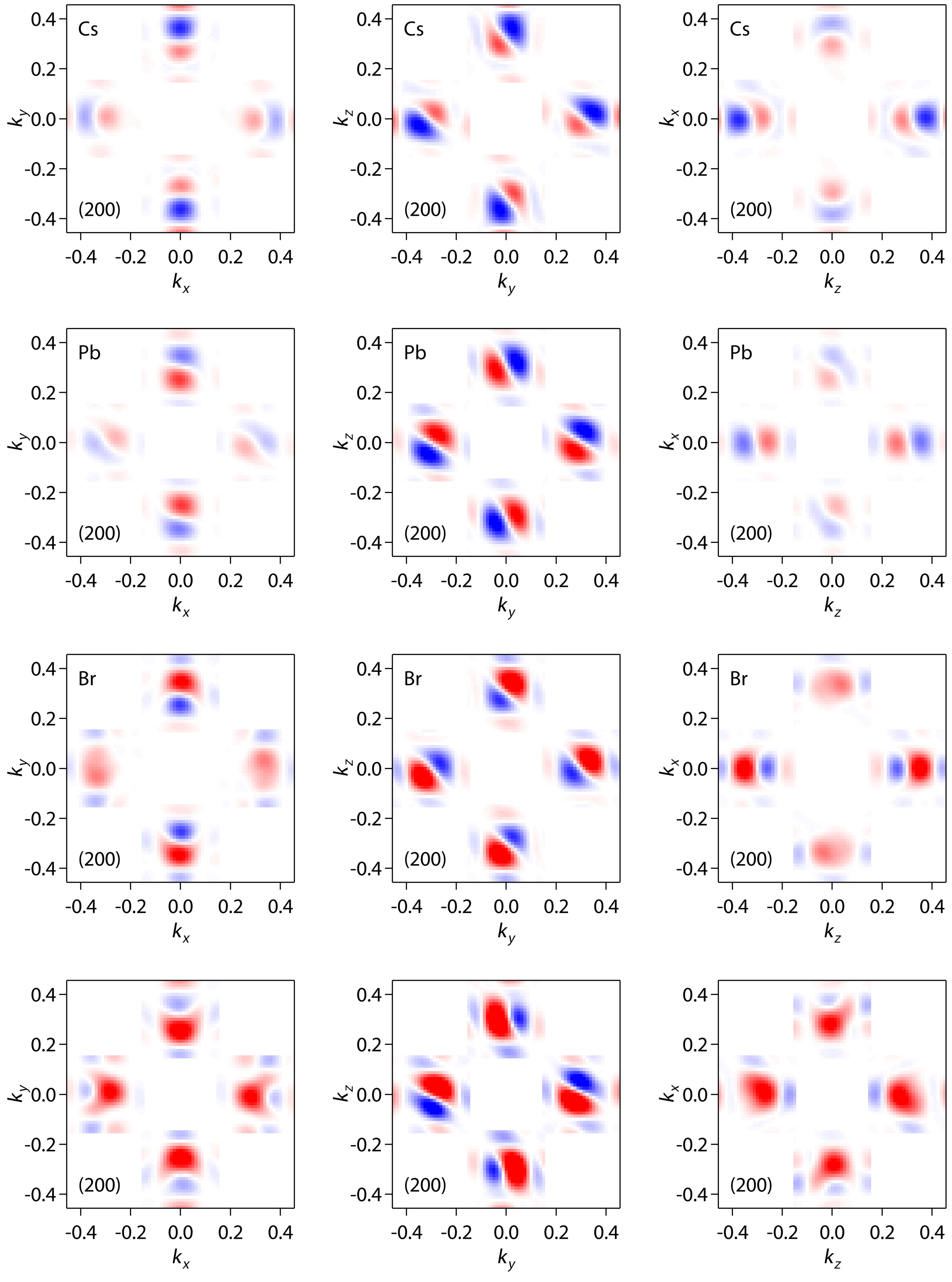

FIG. S12. (3 top rows) Atom-selective diffraction intensity difference maps around the (200) peak and (bottom row) Total diffraction intensity difference around the (200) peak for the box NC.

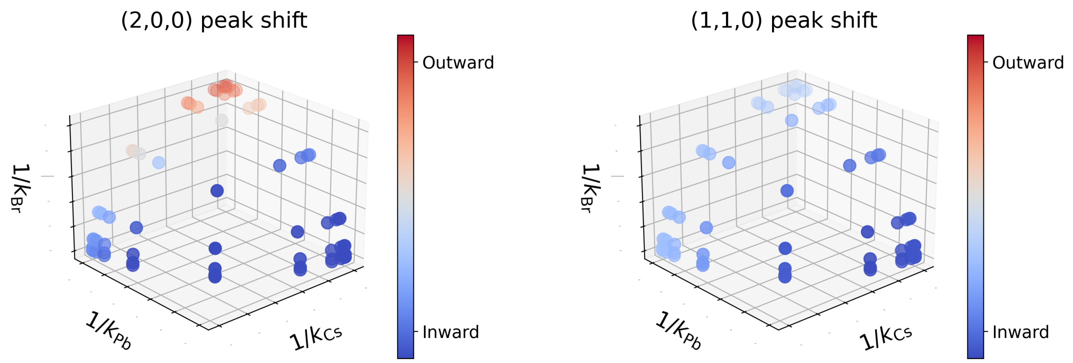

FIG. S13. Peak shifts as a function of restoring force constants with a centrally localized hole charge. Each plot shows the peak shift magnitudes for different atomic restoring force strengths. Red and blue points indicate outward and inward shifts, respectively. In the experimental data, the 200 peak shifts inward and the 110 peak shifts outward upon optical pumping.
